# Plant population and soil origin effects on rhizosphere nematode community composition of a range-expanding plant species and a native congener

**DOI:** 10.1007/s00442-020-04749-y

**Published:** 2020-10-03

**Authors:** Rutger A. Wilschut, Kim J. H. Magnée, S. Geisen, W. H. van der Putten, O. Kostenko

**Affiliations:** 1 Department of Terrestrial Ecology, Netherlands Institute of Ecology (NIOO-KNAW), Wageningen, The Netherlands; 2 Ecology Group, Department of Biology, University of Konstanz, Konstanz, The Netherlands; 3 Laboratory of Plant Breeding, Wageningen University and Research, Wageningen, The Netherlands; 4 Laboratory of Nematology, Wageningen University and Research, Wageningen, The Netherlands

**Keywords:** Enemy release hypothesis, Plant-pathogenic nematodes, Range-expanding plant species, Root-feeding nematodes, Shifting defence hypothesis

## Abstract

Climate change causes species range expansions to higher latitudes and altitudes. It is expected that, due to differences in dispersal abilities between plants and soil biota, range-expanding plant species will become associated with a partly new belowground community in their expanded range. Theory on biological invasions predicts that outside their native range, range-expanding plant species may be released from specialist natural enemies, leading to the evolution of enhanced defence against generalist enemies. Here we tested the hypothesis that expanded range populations of the range-expanding plant species *Centaurea stoebe* accumulate fewer root-feeding nematodes than populations from the original range. Moreover, we examined whether *Centaurea stoebe* accumulates fewer root-feeding nematodes in expanded range soil than in original range soil. We grew plants from three expanded range and three original range populations of *C. stoebe* in soil from the original and from the new range. We compared nematode communities of *C. stoebe* with those of *C. jacea,* a congeneric species native to both ranges. Our results show that expanded range populations of *C. stoebe* did not accumulate fewer root-feeding nematodes than populations from the original range, but that *C. stoebe,* unlike *C. jacea,* accumulated fewest root-feeding nematodes in expanded range soil. Moreover, when we examined other nematode feeding groups, we found intra-specific plant population effects on all these groups. We conclude that range-expanding plant populations from the expanded range were not better defended against root-feeding nematodes than populations from the original range, but that *C. stoebe* might experience partial belowground enemy release.

**Electronic supplementary material:**

The online version of this article (10.1007/s00442-020-04749-y) contains supplementary material, which is available to authorized users.

## Introduction

Current climate change affects the composition of terrestrial and aquatic communities worldwide by causing altitudinal and latitudinal range expansions of plant and animal species within continents (Chen et al. 2011; Pinsky et al. 2020; Steinbauer et al. 2018). As a result of these intracontinental range expansions, terrestrial communities of plants and co-evolved aboveground and belowground organisms are likely to become re-assembled due to differences in dispersal abilities and habitat requirements between species (Berg et al. 2010). In general, soil organisms are expected to be more dispersal-limited than plants, and co-evolved interactions between plants and soil organisms may, therefore, become disrupted (Álvarez-Garrido et al. 2019; Berg et al. 2010). Consequently, plant species that are expanding their range will encounter different soil communities in the new compared to the original range (Ramirez et al. 2019; Wilschut et al. 2019a). Whether these soil communities from the original and new range are functionally different in terms of their impact on plant performance still remains poorly understood (Ramirez et al. 2019; Van Nuland et al. 2017; Wilschut et al. 2019a).

The ecological responses of range-expanding plant species (or neonatives; Essl et al. 2019) to aboveground enemies and feedbacks with soil biota may be quite comparable to responses of intercontinentally introduced exotic plant species (or *aliens*) (Engelkes et al. 2008). Therefore, the large body of literature on exotic plant species can provide a framework for developing and testing of hypotheses on the ecology of neonatives in their new range. For example, there is ample evidence that invasive exotic plant species can have an increased performance in their new range due to the release from co-evolved aboveground and belowground antagonists (‘enemy release hypothesis’; Callaway et al. 2004; Keane and Crawley 2002; Reinhart et al. 2003). Indeed, a number of intracontinental range-expanding plant species have been shown to perform better in soil from the new than from the original range (van Grunsven et al. 2010; Van Nuland et al. 2017). The results of these plant–soil interactions of range-expanding plant species suggest that enemy release may also take place during intracontinental range expansions.

Differences in soil community effects on range-expanding plant performance between the original and new range may possibly also arise upon evolution in the defences of non-native plant populations against plant enemies from the new range. Some non-native plant populations have evolved increased investment in the defence against generalist enemies, in response to the loss of specialist enemies from their original range. Evidence for this ‘shifting defence hypothesis’ mainly comes from studies on aboveground plant–herbivore interactions of intercontinentally introduced exotic plant species (Doorduin and Vrieling 2011; Lin et al. 2015; Zhang et al. 2018). However, it is unknown whether the biotic changes that intracontinental range-expanding plant species experience are strong enough to initiate such evolutionary shifts in defence (Huberty et al. 2014). Likewise, it is unknown whether such evolutionary shifts can take place in the allocation of defence against belowground enemies. Interestingly, recent studies have shown several range-expanding plant populations from the new range and original range to differ in their responses to soil communities and aboveground herbivores (Dostálek et al. 2015; Macel et al. 2017). Whether these differences between populations from the native and new range have evolved locally, or originated from selection against certain genotypes from the native range is difficult to determine. Nevertheless, it is becoming more clear that ecological differences between populations from the original and expanded range can develop rapidly (De Frenne et al. 2014; Lustenhouwer et al. 2018).

So far, little is known about possible differences in belowground community composition between original and new ranges of range-expanding plant species (Ramirez et al. 2019), and their effects on plant performance. One of the soil organism groups potentially affecting range-expanding plant performance are nematodes. Nematodes are the most abundant animals on earth (van den Hoogen et al. 2019) and belong to a functionally diverse group of metazoans containing herbivorous, bacterivorous, fungivorous and predatory or omnivorous taxa. However, despite the strongly negative economic impacts of root-feeding nematodes in agriculture (Nicol et al. 2011), their role in natural systems has remained poorly explored. There is some evidence that the effects of root-feeding nematode accumulation may at least partly underlie plant performance of non-crop species (De Deyn et al. 2003; Wilschut et al. 2019b). While non-herbivorous functional groups of nematodes do not directly interact with plants, they may affect plant performance indirectly via trophic interactions with other groups in the rhizosphere microbiome (Thakur and Geisen 2019). Therefore, changes in the nematode community composition between the original and new range of range-expanding plant species may be of considerable importance for range-expanding plant performance.

Previous comparisons of nematode community composition between range-expanding and native plant species in soils from the new range have shown that range-expanding plant species on average accumulate fewer root-feeding nematodes in their rhizospheres (Morriën et al. 2012; Wilschut et al. 2017). However, there is a high level of plant species-specificity in this accumulation (Wilschut et al. 2017). The ability of root-feeding nematodes from the native range to successfully exploit range-expanding plant species likely relates to the ecological similarity of these range expanders to plant species in the native community. The range-expanding plant species *Centaurea stoebe* has been shown to accumulate hardly any generalist root-feeding nematodes from its new range (Wilschut et al. 2017), which may be caused by its distinct root metabolome (Kulkarni et al. 2018). Possibly, this plant species benefits from defence compounds that do not occur in plant species from the invaded community (‘novel weapon hypothesis’; Callaway et al. 2008; Schaffner et al. 2011). A latitudinal transect study indicated that *Centaurea stoebe* indeed accumulates fewer individuals of certain root-feeding nematode types in its new compared to its original range (Wilschut et al. 2019a). These results suggest a partial release from root-feeding nematodes during range expansion. However, soil and plant effects on nematode community composition can only be fully disentangled in an experimental study.

Here, we examined nematode community composition in the rhizosphere of plants from northern and southern European populations of *Centaurea stoebe*, in soil from both its original and new ranges. As in the new range *C. stoebe* has a strong defence against generalist root-feeding (Wilschut et al. 2017), this species is an interesting candidate to test whether this defence is more pronounced in new range populations than in original range populations. We compared these nematode communities with communities that developed in the root zone of *Centaurea jacea,* a congeneric species that is native throughout Europe, in both the original and new ranges of *C. stoebe*. We explored proportional and total abundances of the main nematode feeding groups, as well as abundances of different types of root-feeding nematodes. We tested the hypotheses that (1) northern populations of *C. stoebe,* but not of *C. jacea,* accumulate fewer root-feeding nematodes than southern populations and (2) root-feeding nematode accumulation by *C. stoebe* is lower in new range soil than in original range soil. The experimental setup, with multiple populations per species from both southern and northern Europe, also allowed us to explore variation in nematode community composition among plant populations, something that has rarely been done. Finally, we examined whether there was co-variation in abundances of nematode feeding groups, and whether abundances of nematode feeding group corresponded to root and shoot biomass variation.

## Methods

### Plant species

We tested our hypotheses using populations of the range-expanding plant species *Centaurea stoebe* (Asteraceae), which originates from central and eastern Europe and has been expanding its range into north-western Europe. Populations of *C. stoebe* have continuously been present in The Netherlands from the beginning of the twenty first century, and since then, the species has steadily become more widespread (NDFF 2019). We also used populations of a native congener of *C. stoebe* that is native throughout Europe: *Centaurea jacea*.

### Seed collection and germination-treatment

For both plant species, seeds were collected from three populations in Slovenia, covering part of the native range of *C. stoebe*, and three populations in the Netherlands, which is the expanded range of *C. stoebe* (population details listed in Supplementary Table 1). Seeds from each population were germinated separately after surface-sterilizing for three minutes in a 10% household bleach solution, after which they were rinsed with demineralized water. The seeds were sown on sterilized glass beads, and germinated under controlled conditions (16/8 h light/dark, 20/10 °C day/night temperature, 60% humidity).

### Soil collection

In both Slovenia and the Netherlands, soils were collected from riverine grassland areas, where both species occur. In the Netherlands, we collected soil from three grassland sites along the river Waal (surroundings of N51° 51′ 32.280′’ E005° 53′ 07.980′’). This soil was used to create a sterilized background soil, and a portion from each of the three sites was kept unsterilized to serve as the ‘new range’ inoculum soil. In Slovenia, ‘original range’ inoculum soil was collected from three distinct areas (N46° 08′ 08.124′’ E014° 36′ 34.992′’; N46° 09′ 54.972′’ E014° 45′ 20.340′’; N45° 58′ 08.544′’ E014° 32′ 44.592′’). For both the new and the original range soils, the three subsamples were combined into single inoculum soils, as we were interested in variation among plant populations within and between the original and new ranges, and not in variation among soil samples within range. All soil was sieved through 5 mm meshes to remove coarse fragments, macro-invertebrates and earthworms. Sterilized background soil was created by autoclaving Dutch soil using a high-pressure saturated stream at 121 °C for 20 to 40 min, depending on the loading weight.

### Experimental setup

In the experiment, five replicate plants of all populations were grown individually in both original range and new range soil in a climate-controlled greenhouse compartment (16/8 h light/dark, 25/15 °C day/night temperature, 60% humidity), resulting in 120 experimental units (2 species × 2 plant origins x 3 populations x 2 soils x 5 replicates). Pots of 1L were filled with a mixture of 90% sterilized Dutch soil and 10% of either alive new range soil or original range soil. Two weeks after the plant seeds were sown on glass beads a single, randomly-selected seedling was transplanted to each pot. After 6 weeks of growth, the pots were placed in mesh cages to serve as control treatment for other plants treated with aboveground herbivorous insects (data not shown). After 11 weeks of growth, plants were harvested by clipping, drying and weighing the aboveground parts. Roots were washed, dried and weighed. Per pot, 100 g of soil was collected and stored at 4 °C until nematode extraction. Additionally, for each pot a soil sample was dried to estimate moisture content, which was later used to standardize nematode numbers to N/100 g dry soil.

### Nematode extraction & identification

Nematodes were extracted from the soil samples using an Oostenbrink elutriator (Oostenbrink 1960). Thereafter, nematode suspensions were concentrated to 10 ml, after which nematode counting and identification was performed using an inverted light microscope (Olympus CK40; 40–400 × magnification). All nematodes in these samples were counted and, based on their morphological characteristics, assigned to four different feeding groups: root feeders, fungivores, bacterivores and predators/omnivores. Root-feeding nematodes were further identified to family or genus-level. We classified the root-feeding nematodes as families or genera: Anguinidae, Criconomatidae, Dolichodoridae, Hoplolaimidae, Tylenchidae, *Meloidogyne* (Heteroderidae), *Paratylenchus* (Tylenchulidae) and *Pratylenchus* (Pratylenchidae). The detection of Dolichodoridae in original range soil samples was very difficult due to the presence of an abundant fungivorous nematode, *Tylencholaimellus sp.,* which strongly resembles members of the Dolichodoridae family. Therefore, a small number of Dolichodoridae may have been overlooked in original range soil samples. Based on Yeates et al. (1993), we assigned these root-feeding nematode taxa to four different root-feeding nematode types: endoparasites (*Meloidogyne*, *Pratylenchus*), semi-endoparasites (Hoplolaimidae), ectoparasites (Criconomatidae, Dolichodoridae, *Paratylenchus*) and root-hair feeders (Tylenchidae). Nematodes in the family Anguinidae cannot be classified to a single feeding-type and were left out of this root-feeding nematode feeding-type analysis. Our identification approach did not allow us to determine whether root-feeding nematode taxa were specialists or generalists, and therefore, we could not determine possible changes in accumulation of generalist versus specialist root-feeding nematodes. However, root-feeding nematodes from diverse grassland systems are thought to be mostly generalists (Van der Putten 2003), making our study system relevant to examine possible changes against generalist root-feeding nematodes.

### Statistical analyses

All statistical analyses were performed in R version 3.6.1 (Team 2013). To examine variation in root and shoot biomass we ran general linear models with the fixed factors ‘plant species’ (levels: C. stoebe/C. jacea), ‘population origin’ (levels: north/south) and ‘soil’ (levels: north/south) with all possible interactions. Then, for root and shoot biomass of each plant species separately, models were run with fixed factors ‘population’ (levels: north1/north2/north3/south1/south2/south3), ‘soil’ and the ‘population*soil’ interaction. We analysed the total and proportional abundance of root feeders, bacterivores, fungivores and the combined group of predators and omnivores. For total abundances we used generalized linear models with a negative binomial distribution and a log link function (glm.nb in *MASS*), while for the proportional abundances we ran general linear models (lm in *STATS*). For all these nematode response groups we first ran models containing the fixed factors ‘plant species’, ‘population origin’ and ‘soil’ with all possible interactions. To gain insight into population and population origin effects within species, we also ran models separately for each plant species, containing either the factors ‘population origin’, ‘soil’ and ‘population origin*soil’, or ‘population’, ‘soil’ and ‘population*soil’. Similar analyses were performed for total abundances of the different types of root-feeding nematodes. Additionally, we analysed root-feeding nematode abundances adjusted for root biomass (N/100 g soil g root^−1^) with a generalized linear model with negative binomial distribution with the fixed factors plant species’, ‘population origin’ and ‘soil’ with all possible interactions. Finally, we used Pearson correlation tests to examine whether numbers of the different nematode feeding groups could be explained by root weight and by abundances of other feeding groups. We performed these tests separately for each *Centaurea* species, both for the combination of northern and southern soil, as well as separately for each of the two soils.

## Results

### Nematode counts and plant biomass

**Fig. 1:**
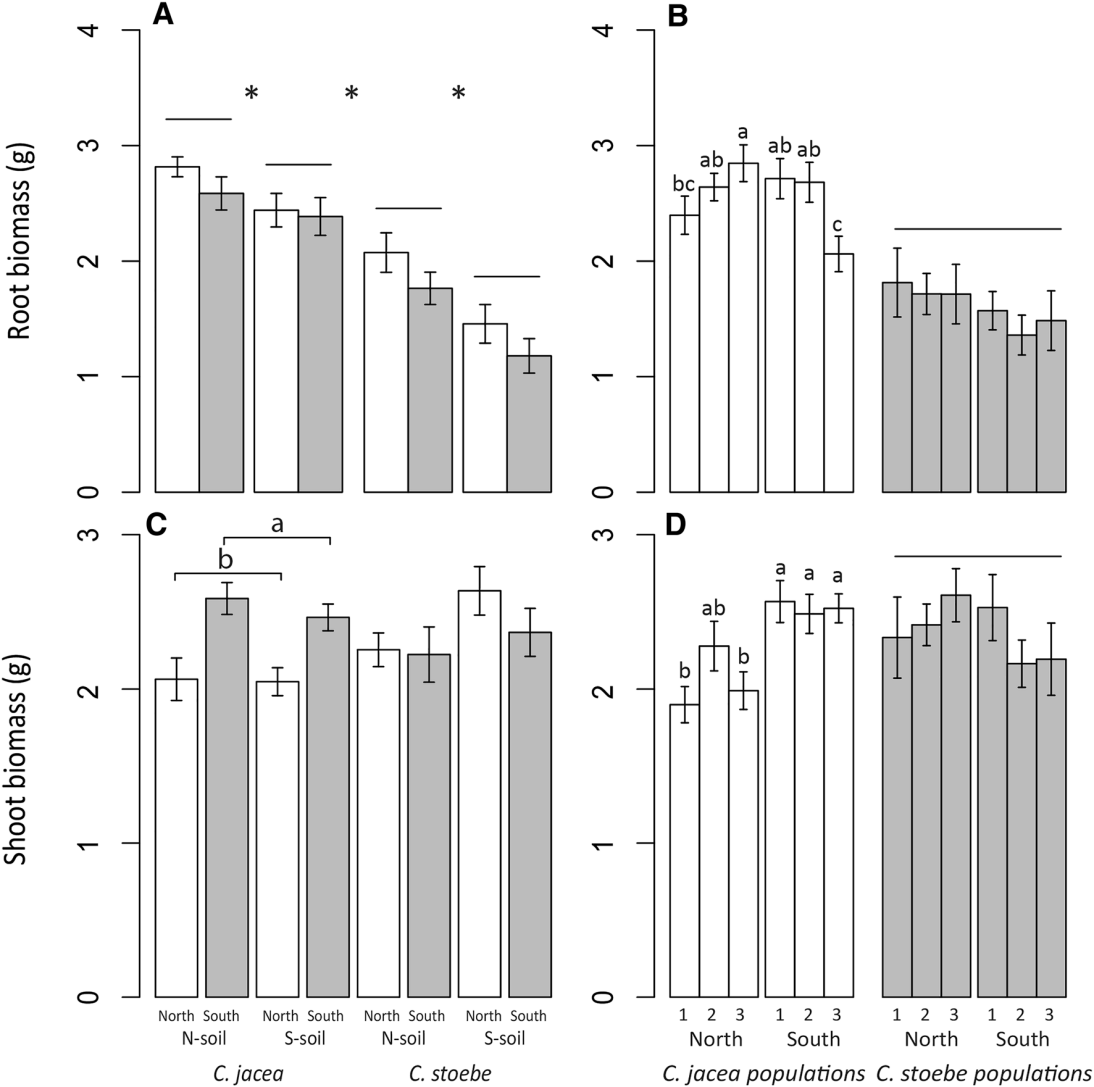
**A**, **C** Root and shoot biomass (g) of plants from northern (white) and southern populations (grey) of native *Centaurea jacea* and range-expanding *Centaurea stoebe*, in northern (N) and southern (S) soil, and **B**, **D** root and shoot biomass of individual populations from northern (1,2,3) and southern (1,2,3) origin of *C. jacea* (white) and *C. stoebe* (grey), averaged over northern and southern soil. Bars represent means ± standard errors. Asterisks (*) indicate significant differences between species and between soils within species. Horizontal lines indicate the absence of significant differences between groups of populations or individual populations, while small letters indicate significant differences between populations based on post hoc analysis

Total nematode numbers (N/100 g^−1^) ranged from 67 to 1889 per sample, with on average 645 nematodes in *Centaurea jacea* samples and 823 in *Centaurea stoebe* samples. Root systems of *C. jacea* were bigger than those of *C. stoebe* (F = 85.7, *p* < 0.001; Fig. 1A), and both species grew larger root systems in northern soil than in southern soil (F = 17.73, *p* < 0.001; Fig. 1A). Irrespective of plant species, individuals from northern populations on average tended to have bigger root systems than individuals from southern populations (F = 3.86, *p* = 0.052; Fig. 1A). Root weight significantly differed among populations of *C. jacea* (F = 3.50, *p* < 0.01; Fig. 1B), but not among populations of *C. stoebe* (F = 0.69, *p* = 0.63; Fig. 1B), and in both species, variation in root weight between populations did not depend on soil. Plants from southern populations of *C. jacea* developed more shoot biomass than plants from northern populations, while there was no significant shoot biomass variation within *C. stoebe* (species*plant origin; F = 11.35, *p* < 0.01; Fig. 1C). Shoot weight significantly differed among populations of *C. jacea* (F = 4.62, *p* < 0.01; Fig. 1D), but not among populations of *C. stoebe* (F = 0.91, *p* = 0.48; Fig. 1D). Soil origin did not affect shoot biomass in any analysis.

### Nematode abundances

**Fig. 2:**
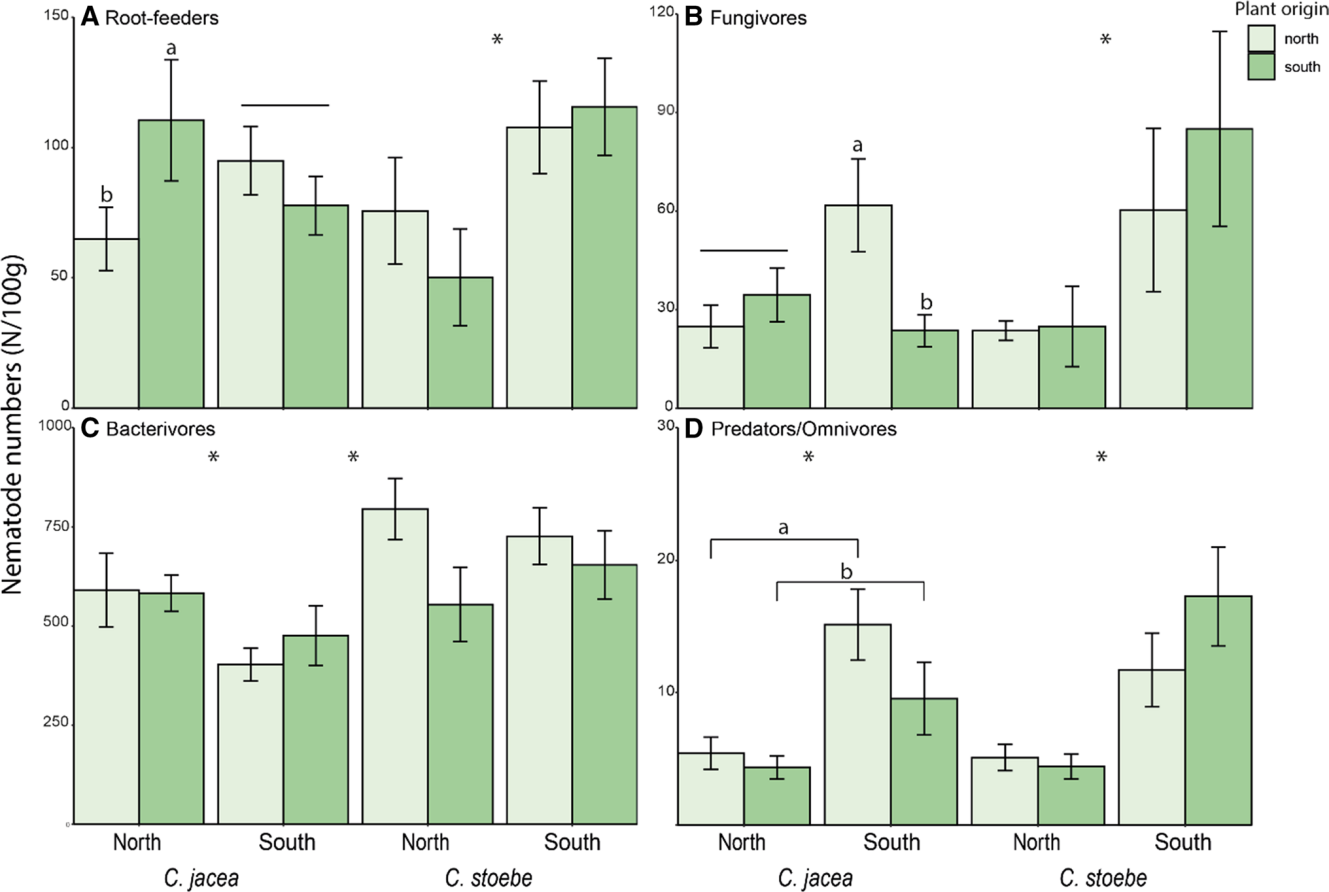
Absolute abundances of the four main nematode feeding groups in plants from northern (light green) and southern populations (dark green) of the native plant species *C. jacea* and the range-expanding plant species *C. stoebe*, in northern and southern soil. Bars represent means ± standard errors. Asterisks (*) indicate significant differences between species or between soils within species. Horizontal brackets with small letters indicate differences between populations of different origins. Combinations of horizontal lines and small letters indicate post hoc analyses of within-species interactions between plant origin and soil

**Fig. 3:**
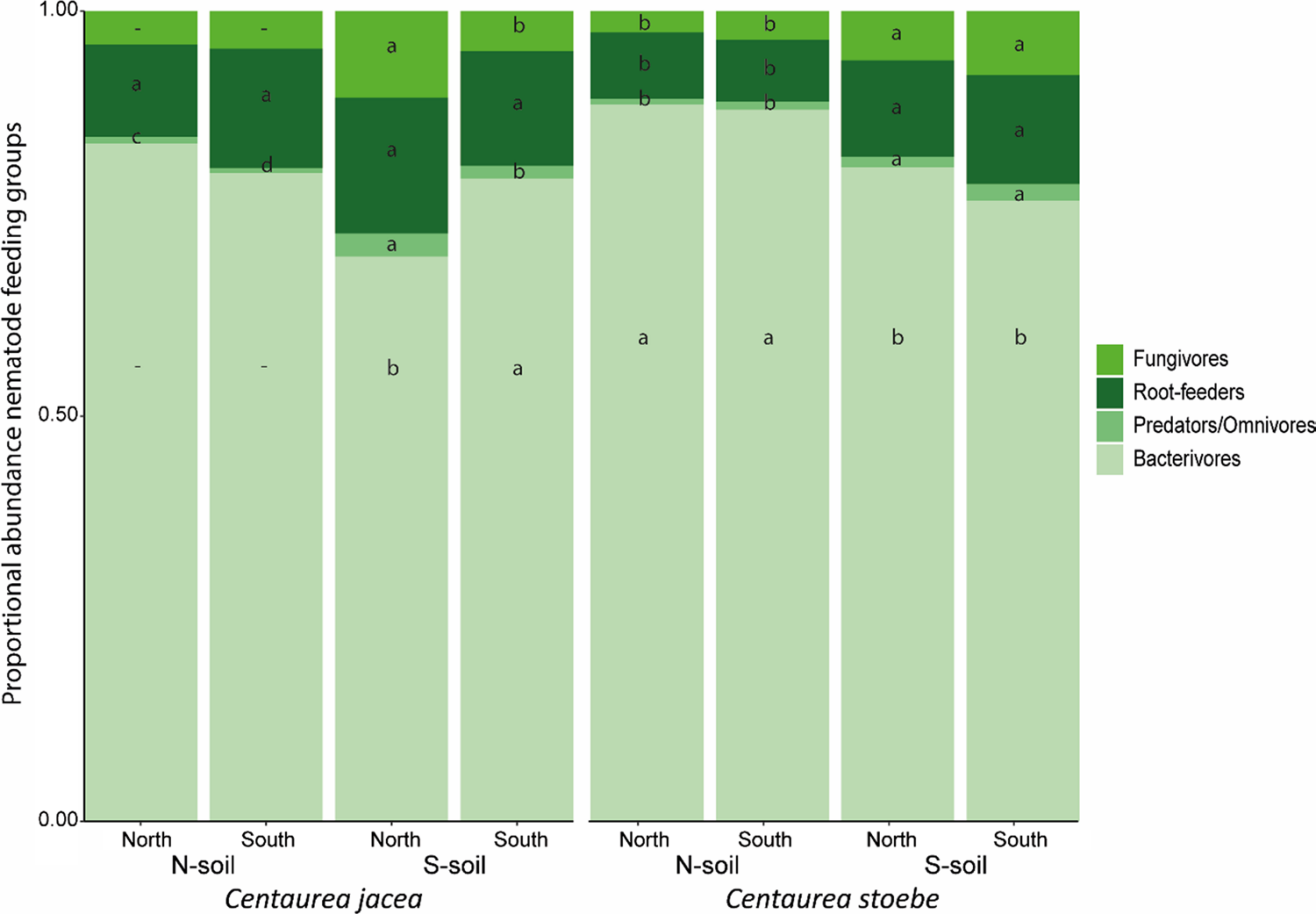
Proportional abundances of the four main nematode feeding groups in plants from northern and southern populations of native plant species *C. jacea* and range-expanding plant species *C. stoebe*, in northern (N) and southern (S) soil. Per nematode feeding group, small letters indicate within-species differences between soils or population origins, while the combinations of dashes (−) and small letters indicate the results of within-species interactions between population origin and soil

Root feeders: total numbers of root-feeding nematodes were explained by the three-way interaction between species, soil and origin of the plants (Table 1). The range-expander *Centaurea stoebe* accumulated fewer root-feeding nematodes in northern soils than in southern soils (Table 2, Fig. 2). In the case of native *C. jacea*, plants from southern populations accumulated more root-feeding nematodes than plants from northern populations, but only in northern soil (Table 2, Fig. 2). In both *Centaurea* species variation in numbers of root-feeding nematodes among populations tended to depend on soil (marginally significant effects; Table 2, Fig. S1). The proportion of root-feeding nematodes in the nematode community was higher in pots with *C. jacea* than with the range-expander *C. stoebe*, and was highest in southern soils (Table 1, Fig. 3). Separate analyses per plant species indicated that *Centaurea stoebe* had the lowest proportion of root-feeding nematodes in northern soils, whereas there was no such difference in the case of *C. jacea* (Table 2, Fig. 3). The proportion of root-feeding nematodes tended to differ among populations for *C. jacea* (marginally significant effect), but not in the case of *C. stoebe* (Table 2, Fig. S2). *Centaurea stoebe* accumulated higher root-feeding nematode densities per gram of root in southern than in northern soil, while *C. jacea* accumulated comparable densities in both soils (species*soil: explained deviance = 10.89, *p* < 0.001; Fig. S3). Averaged over species, populations from southern origin accumulated higher root-feeding nematode densities per gram root than populations from northern origin (origin effect: explained deviance = 3.92, *p* < 0.05; Fig. S3).

**Table 1:** Model results of negative-binomial generalized linear models (absolute abundances; above) and general linear models (proportional abundances; below) modelling the abundances of root-feeding, bacterivorous, fungivorous and predatory-omnivorous nematodes

		Species (Sp)		Origin (O)		Soil (S)		Sp*O		Sp*S		O*S		Sp*O*S	
		Dev.	*p*	Dev.	*p*	Dev.	*p*	Dev.	*p*	Dev.	*p*	Dev.	*p*	Dev.	*p*
*Abs. abundance*	Root feeders	0.00	0.96	0.05	0.82	**4.91**	**< 0.05**	1.97	0.16	**5.58**	**< 0.05**	0.34	0.56	**5.86**	**< 0.05**
	Fungivores	3.28	0.07	0.28	0.60	**15.73**	**< 0.001**	1.36	0.24	**5.32**	**< 0.05**	2.19	0.14	**5**.**06**	**< 0.05**
	Bacterivores	**8.43**	**< 0.01**	0.66	0.42	1.78	0.18	2.38	0.12	3.19	0.07	1.30	0.25	0.04	0.84
	Predators/Omnivores	0.71	0.40	0.34	0.56	**39.38**	**< 0.001**	2.47	0.12	0.41	0.52	0.16	0.69	1.42	0.23

		*F*	*p*	*F*	*p*	*F*	*p*	*F*	*p*	*F*	*p*	*F*	*p*	*F*	*p*
*Prop. abundance*	Root feeders	**9.18**	**< 0.01**	0.07	0.79	**7.70**	**< 0.01**	0.00	0.96	0.86	0.36	0.64	0.43	2.41	0.12
	Fungivores	0.73	0.39	0.47	0.49	**10.87**	**< 0.01**	3.26	0.07	0.06	0.81	1.63	0.20	2.64	0.11
	Bacterivores	**8.04**	**< 0.01**	0.09	0.76	**22.44**	**< 0.001**	2.36	0.13	0.43	0.51	2.14	0.15	**5.59**	**< 0.05**
	Predators/Omnivores	0.47	0.50	0.27	0.60	**24.68**	**< 0.001**	**7.81**	**< 0.01**	1.65	0.20	0.42	0.52	2.73	0.10

Included factors are plant species (Sp), plant origin (O), and soil (S), and all possible interactions. *F* values (general linear models) or explained deviance (Dev.; negative binomial models) and *p* values are shown. Significant results (*p* < 0.05) are marked in bold

**Table 2:** Per species results of general linear models (absolute abundances; above) and negative-binomial generalized linear models (proportional abundances; below) modelling the abundances of root-feeding, bacterivorous, fungivorous and predatory-omnivorous nematodes. Included factors are soil (S), plant origin (O), population (P) and the origin*soil and population*soil interactions

		Origin-model						Population-model					
		Origin (O)		Soil (S)		O*S		Population (P)		Soil (S)		P*S	
		Dev.	*p*	Dev.	*p*	Dev.	*p*	Dev.	*p*	Dev.	*p*	Dev.	*p*
*Absolute abundance*													
*C. jacea*	Root feeders	1.03	0.31	0.01	0.94	**5.13**	**< 0.05**	**22.60**	**< 0.001**	0.03	0.87	*10.98*	*0.052*
	Fungivores	*3.50*	*0.06*	1.45	0.23	**8.97**	**< 0.01**	8.79	0.12	1.12	0.29	**20.72**	**< 0.001**
	Bacterivores	0.22	0.64	**4.64**	**< 0.05**	0.44	0.51	**18.02**	**< 0.01**	**5.04**	**< 0.05**	3.08	0.69
	Predators/Omnivores	2.49	0.11	**12.88**	**< 0.001**	0.23	0.63	8.61	0.13	**12.73**	**< 0.001**	2.31	0.80
*C. stoebe*	Root feeders	0.36	0.55	**9.78**	**< 0.01**	1.55	0.21	*10.77*	*0.06*	**12.78**	**< 0.001**	*10.56*	*0.06*
	Fungivores	0.70	0.40	**14.39**	**< 0.001**	0.26	0.61	4.88	0.43	**15.51**	**< 0.001**	**18.57**	**< 0.01**
	Bacterivores	2.94	0.09	0.13	0. 72	0.93	0.34	**15.02**	**< 0.05**	0.05	0.82	9.02	0.11
	Predators/Omnivores	1.21	0.27	**27.75**	**< 0.001**	1.59	0.21	**13.27**	**< 0.05**	**32.53**	**< 0.001**	2.89	0.72

		Origin (O)		Soil (S)		O*S		Population (P)		Soil (S)		P*S	
		*F*	*p*	*F*	*p*	*F*	*p*	*F*	*p*	*F*	*p*	*F*	*p*
*Proportional abundance*													
*C. jacea*	Root feeders	0.03	0.87	1.45	0.23	2.16	0.15	*2.35*	*0.05*	1.62	0.21	1.11	0.37
	Fungivores	3.14	0.08	**5.37**	**< 0.05**	**4.52**	**< 0.05**	1.03	0.41	**5.30**	**< 0.05**	1.94	0.10
	Bacterivores	1.22	0.27	**7.27**	**< 0.01**	**5.97**	**< 0.05**	*2.31*	*0.06*	**8.40**	**< 0.01**	**2.69**	**< 0.05**
	Predators/Omnivores	**4.07**	**< 0.05**	**16.35**	**< 0.001**	2.09	0.15	1.58	0.18	**15.79**	**< 0.001**	0.83	0.54
*C. stoebe*	Root feeders	0.05	0.83	**9.44**	**< 0.01**	0.45	0.51	0.37	0.87	**9.45**	**< 0.01**	1.32	0.27
	Fungivores	0.56	0.46	**5.73**	**< 0.05**	0.08	0.78	0.30	0.91	**5.27**	**< 0.05**	0.46	0.80
	Bacterivores	0.90	0.35	**18.64**	**< 0.001**	0.62	0.44	0.43	0.83	**17.51**	**< 0.001**	0.72	0.61
	Predators/Omnivores	3.39	0.07	**8.87**	**< 0.01**	0.75	0.39	2.31	0.06	**10.22**	**< 0.01**	1.38	0.25

*F* values (general linear models) or explained deviance (Dev.; negative binomial models) and *p* values are shown. Significant results (*p* < 0.05) are marked in bold, marginally significant results (0.05 > *p* < 0.07) in italic

**Fig. 4:**
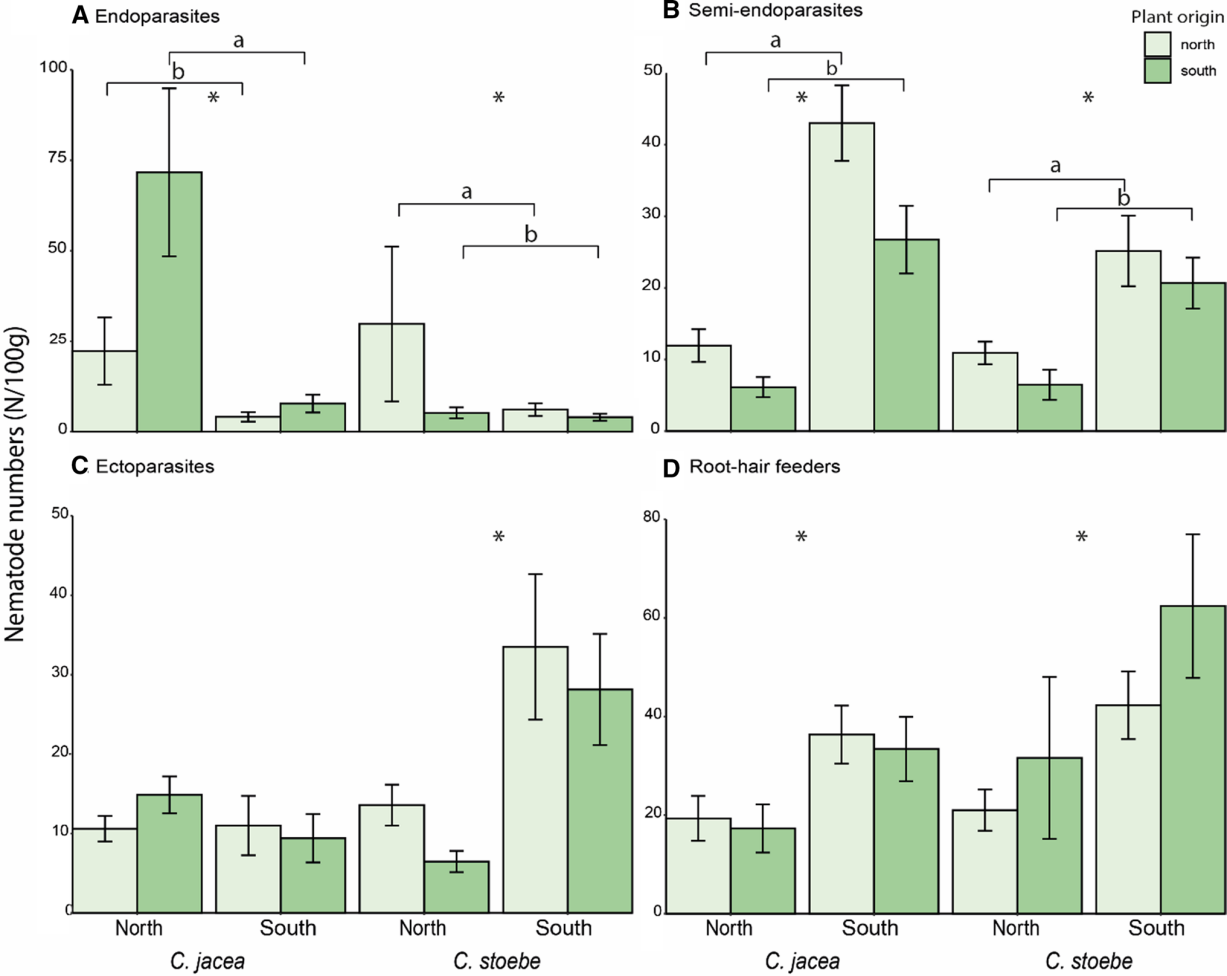
Absolute abundances of the four main root-feeding nematode types in plants from northern (light green) and southern populations (dark green) of native plant species *C. jacea* and range-expanding plant species *C. stoebe*, in northern and southern soil. Bars represent means ± standard errors. Asterisks (*) indicate significant differences between soils within species. Horizontal brackets with small letters indicate differences between different plant origins based on post hoc analysis

Root-feeding nematode types: Effects of plant species, soil and plant origin on root-feeding nematode abundances were not uniform among the different types of root-feeding nematodes. Both *Centaurea* species accumulated most endoparasites in northern soils (Table 3, Fig. 4). On average, northern populations of *C. stoebe* accumulated more endoparasites than southern populations, while the opposite was found in *C. jacea* (Table 3, Fig. 4). Populations of both *Centaurea* species varied in their accumulation of endoparasites, and this variation depended on soil origin in the case of *C. stoebe* (Table 3, Fig. S4). Semi-endoparasite numbers were higher in southern soils than in northern soils (Table 3, Fig. 4). In *C. jacea,* plants from northern populations accumulated more semi-endoparasites than plants from southern populations, while in *C. stoebe* there was overall variation in semi-endoparasite accumulation among populations (Table 3, Fig. 4). *Centaurea stoebe* accumulated significantly fewer ectoparasites in northern soils than in southern soils, whereas ectoparasite numbers did not differ between soils in the case of *C. jacea* (Table 3, Fig. 4). In *C. jacea,* variation in ectoparasite accumulation among populations depended on the soil, while no variation among populations was found in *C. stoebe* (Table 3, Fig. S4). Irrespective of plant species, root-hair feeder numbers were higher in southern soils than in northern soils and in both plant species there was significant variation in numbers of root-hair feeders among populations (Table 3, Fig. 4, Fig. S4).

**Table 3:** Per species results of negative-binomial generalized linear models modelling the absolute abundances of four root-feeding nematode groups: endoparasites, semi-endoparasites, ectoparasites and root-hair feeders (see “Methods” Section)

		Origin-model						Population-model					
		Origin (O)		Soil (S)	O*S			Population (P)		Soil (S)		P*S	
		Dev.	*p*	Dev.	*p*	Dev.	*p*	Dev.	*p*	Dev.	*p*	Dev.	*p*
*C. jacea*	Endoparasites	**11.19**	**< 0.001**	**31.54**	**< 0.001**	0.64	0.42	**40.90**	**< 0.001**	**36.25**	**< 0.001**	4.70	0.45
	Semi-endoparasites	**7.94**	**< 0.01**	**51.88**	**< 0.001**	0.26	0.61	**13.36**	**< 0.05**	**66.67**	**< 0.001**	6.74	0.24
	Ectoparasites	0.22	0.64	0.72	0.40	1.00	0.32	**17.52**	**< 0.01**	**6.90**	**< 0.01**	**22.83**	**< 0.001**
	Root-hair feeders	0.24	0.62	**11.25**	**< 0.001**	0.01	0.94	**30.22**	**< 0.001**	**18.88**	**< 0.001**	**12.49**	**< 0.05**

		Origin (O)		Soil (S)	O*S			Population (P)		Soil (S)		P*S	
		Dev.	*p*	Dev.	*p*	Dev.	*p*	Dev.	*p*	Dev.	*p*	Dev.	*p*
*C. stoebe*	Endoparasites	**14.82**	**< 0.001**	**6.69**	**< 0.01**	*3.83*	*0.05*	**38.12**	**< 0.001**	3.03	0.08	**13.29**	**< 0.05**
	Semi-endoparasites	2.13	0.14	**20.37**	**< 0.001**	0.56	0.46	**21.27**	**< 0.001**	**28.73**	**< 0.001**	1.85	0.87
	Ectoparasites	2.05	0.15	**23.26**	**< 0.001**	1.38	0.24	**11.69**	**< 0.05**	**22.82**	**< 0.001**	9.58	0.09
	Root-hair feeders	2.69	0.10	**9.21**	**< 0.01**	0.00	0.97	**15.85**	**< 0.01**	**13.61**	**< 0.001**	6.88	0.23

Included factors are soil (S), plant origin (O), population (P) and the origin*soil and population*soil interactions. Explained deviance (Dev.) and *p* values are shown. Significant results (*p* < 0.05) are marked in bold, marginally significant results (0.05 > *p* < 0.06) in italic

Fungivores: Total numbers of fungivorous nematodes depended on the three-way interaction between species, soil and origin of the plants (Table 1). *Centaurea stoebe* accumulated fewest fungivorous nematodes in northern soil, although this soil effect varied among populations (Table 2, Fig. 2, Fig. S1). In the case of *C. jacea*, plant origin effects on fungivores depended on the soil they were grown in: in southern soil plants from northern populations accumulated more fungivores than plants from southern populations, while this was not the case in northern soil (Table 2, Fig. 2). Irrespective of plant species, southern soils contained the highest proportion of fungivorous nematodes (Table 1). In *C. jacea,* proportional abundances of fungivores also depended on interaction between soil and plant origin as was the case for total numbers (Table 2, Fig. 3).

Bacterivores: *Centaurea stoebe* overall accumulated more bacterivorous nematodes than *C. jacea* (Table 1, Fig. 2). *Centaurea jacea* plants on average accumulated more bacterivores in northern soils than in southern soils (Table 1, Fig. 2). Moreover, northern populations of *C. stoebe* accumulated more bacterivores than plants from southern populations. Variation in numbers of bacterivores among plant populations was significant in both *Centaurea* species (Table 2). Proportional abundances of bacterivores depended on the three-way interaction between plant species, plant origin and soil (Table 1). In *C. stoebe*, proportional bacterivore abundances were higher in northern soils than in southern soils, while in *C. jacea* plants from northern populations tended to accumulate lower proportional abundances of bacterivores in southern soils but higher proportional abundances in northern soils (Table 2, Fig. 3).

Predators/Omnivores: Pots with southern soil contained higher total and proportional abundances of predatory-omnivorous nematodes than pots with northern soil (Table 1, Figs. 2, 3). While *C. jacea* plants from northern populations accumulated higher proportional abundances of predatory-omnivorous nematodes than plants from southern populations, the opposite tended to be the case for *C. stoebe* plants (Table 2, Fig. 3). Total numbers of predatory-omnivorous nematode numbers varied significantly among populations in *C. stoebe*, but not in *C. jacea* (Table 2, Fig. S1).

### Correlation tests

**Fig. 5:**
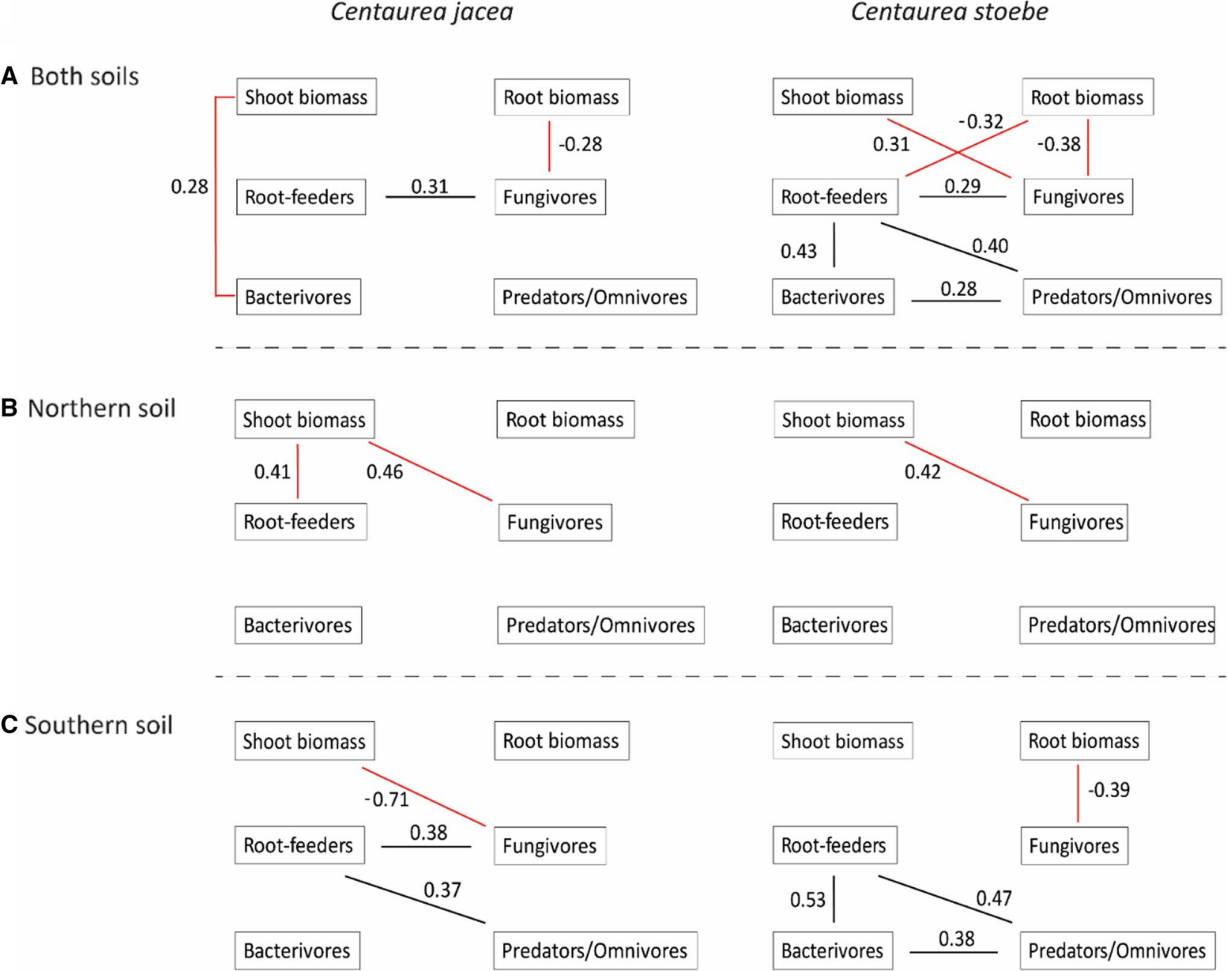
Overview of Pearson correlation tests between root and shoot biomass and nematode feeding group abundance (red lines) and between different nematode feeding groups (black lines) for native *Centaurea jacea* (left) and range-expanding *Centaurea stoebe* (right), in both northern and southern soil combined (**a**), as well as separately for each soil (**b**, **c**). Only significant correlations (*p* < 0.05) are shown

The outcome of correlation tests between plant (root and shoot) biomass and nematode numbers in the four nematode feeding groups, as well as correlations between abundances of the different nematode feeding groups, depended on the plant species and whether the data from both soils were combined or analysed separately (Fig. 5). Overall, root biomass of *C. jacea* correlated negatively with numbers of fungivorous nematodes, and fungivorous nematodes correlated positively with numbers of root-feeding nematodes (Fig. 5). *Centaurea jacea* shoot biomass overall correlated positively with numbers of bacterivorous nematodes. In northern *C. jacea* soil, numbers of fungivorous and root-feeding nematodes correlated positively with shoot biomass, while numbers of fungivores correlated negatively with shoot biomass in southern *C. jacea* soil. In southern *C. jacea* soil, numbers of root-feeding nematodes correlated positively with numbers of fungivores and predatory-omnivorous nematodes (Fig. 5). Root biomass of *C. stoebe* overall was correlated negatively with numbers of fungivorous and root-feeding nematodes, while shoot biomass of *C. stoebe* overall correlated positively with numbers of fungivores. This positive correlation between shoot biomass and numbers of fungivores was also found in northern *C. stoebe* soils, but not in southern *C. stoebe* soils. In southern *C. stoebe* soils, root biomass negatively correlated with numbers of fungivores. Numbers of root-feeding nematodes in *C. stoebe* soils overall positively correlated with numbers of fungivorous, bacterivorous and predatory-omnivorous nematodes, which was also found in southern *C. stoebe* soils, but not in northern *C. stoebe* soils (Fig. 5).

## Discussion

We explored responses of nematode communities from a southern and a northern European soil to populations of *Centaurea jacea*, which is a native plant species across Europe, and *Centaurea stoebe*, which is expanding its range into northern Europe. Studies on introduced exotic species have shown that new range populations of non-native plant species may be better defended against generalist herbivores, at the expense of defence against specialist herbivores (Blossey and Notzold 1995; Doorduin and Vrieling 2011). However, very few studies have been done to test whether original and new range populations of range-expanding plant species differ in their responses to belowground herbivores. In our study we did not find support for our hypothesis that northern populations of the range-expanding plant species accumulate fewer root-feeding nematodes from the new range than southern populations. Possibly, the changes in belowground community composition that *C. stoebe* has experienced during range expansion have not been strong enough to select for genotypes that accumulate fewer root-feeding nematodes due to increased belowground defences (Ramirez et al. 2019; Wilschut et al. 2019a). Our results coincide with a study showing that expanded range populations of the range-expanding plant species *Rorippa austriaca* were not better defended against a widespread aboveground generalist herbivore (Huberty et al. 2014).

In support of our second hypothesis, we found that, in contrast to the native *C. jacea,* the range-expander *Centaurea stoebe* accumulated fewer root-feeding nematodes in the soil from the new range than in the soil from the original range. Moreover, numbers of root-feeding nematodes correlated negatively with root biomass of *C. stoebe*, but not of *C. jacea*. However, these correlations may not necessarily be explained by root-feeding nematode effects on root biomass, as *C. jacea* did not accumulate most nematodes in southern soil, although there its root biomass was lowest. Moreover, aboveground biomass was not affected by soil origin in either of the species, indicating that potential effects of nematode community changes were only reflected in belowground biomass. Nevertheless, considering that *C. stoebe* plants produced the lowest root biomass in the southern soil, where they accumulated most root-feeding nematodes, the actual exposure to root-feeding nematodes per unit of root mass was considerably higher in southern soil than in northern soil (Fig. S3). Our results, therefore, suggest that range-expanding plant species may accumulate fewer root-feeding nematodes in soil from the new range than in soil from the original range. This could partly underlie the enhanced performance of range-expanding plant species in the new range, as has been found in studies so far (De Frenne et al. 2014; van Grunsven et al. 2010; Van Nuland et al. 2017). However, it must be acknowledged that in our study we created two composite soils to represent an original and a new range belowground community. A study with multiple spatially independent soil replicates is, therefore, needed to study root-feeding nematode accumulation between original and new range soils in general (Gundale et al. 2017; van Grunsven et al. 2010).

Previous work on non-native and native populations of the grass species *Ammophila arenaria* has shown that non-native plants may especially be released from endoparasitic root-feeding nematodes (van der Putten et al. 2005). Also in a latitudinal transect study covering both the original and new range of *C. stoebe,* numbers of endoparasites, as well as of semi-endoparasites, were found to be lowest in the new range (Wilschut et al. 2019a). It is, therefore, surprising that in the current study especially the numbers of ectoparasites were reduced in the new range soil. It must be noted that in our current study numbers of endoparasites will have been underestimated, as we only extracted nematodes from soil and not from roots. The taxonomic resolution of nematode identification in both the transect and the current study does not allow to infer whether the observed reductions of root-feeding nematode numbers are caused by a loss of specific taxa from the original range, or by a reduction of nematode numbers from the same taxa. To examine this, future studies should determine whether plant species are associated to specialist root-feeding nematode taxa in their original range, for example using molecular techniques that allow identification of nematodes up to, or even below species-level (Seesao et al. 2017). When such methods are combined with sequencing of other organism groups in the rhizosphere, e.g., bacteria and fungi, also mechanisms underlying changes in the community of non-herbivorous nematodes may be determined. In this way, it might be determined why fungivorous nematodes are reduced in new range soil of *C. stoebe*. Possibly, the root compounds of *C. stoebe* may have inhibited fungal growth in the new range because of their novelty (Morriën and van der Putten 2013; Wilschut et al. 2017). Alternatively, *C. stoebe* may directly have affected fungivorous nematodes from the new range, when this group comprised taxa that facultatively feed on roots, such as species of *Aphelenchoides* or *Aphelenchus* (Yeates et al. 1993).

While our study did not show differences in the abundances of nematode feeding groups between northern and southern populations of the range-expanding *C. stoebe*, northern and southern populations of *C. jacea* did differ in the accumulation of root feeders, as well as fungivores. Although we do not yet understand the underlying mechanisms, the results are in line with previous studies showing that throughout their range, native plant species may show considerable variation in nematode community composition (Wilschut et al. 2019a). Interestingly, in both plant species, northern and southern populations differed in the accumulation of endoparasitic and semi-endoparasitic root-feeding nematodes. In both species the accumulation of relatively high numbers of endoparasites corresponded with low numbers of semi-endoparasites and vice versa. Perhaps, semi-endoparasites may have competitively been suppressed in populations that accumulate relatively high numbers of endoparasites (Brinkman et al. 2004). However, it cannot be ruled out that these populations differ in the group of nematodes against which they are most well-defended.

In addition to differences between plant populations of different geographic origin, our analyses showed that in both plant species there was significant variation in absolute abundances of the majority of the nematode feeding groups as well as the different root-feeding nematode types between populations. Such population or plant genotype effects on the rhizosphere microbiome are well known (Agler et al. 2016; Wagner et al. 2016), but for nematodes these effects have predominantly been shown in agricultural systems (Boerma and Hussey 1992; Palomares-Rius et al. 2012). However, a study on *Ammophila arenaria* showed that natural plant populations can differ in the accumulation of endoparasitic nematodes (de la Pena et al. 2009), as was found in our study as well. Some of the variation in nematode abundances correlated positively with shoot biomass, possibly suggesting stimulation of bacterivorous and fungivorous nematodes via increased rhizodeposition (Dam and Christensen 2015), but these effects differed between plant species and were soil-dependent. In contrast to absolute abundances, relative abundances of the different nematode feeding groups hardly showed variation between plant populations within the same plant species, indicating that while populations differed in their accumulation of nematodes, the structure of their nematode communities are highly similar. This pattern could not be explained by variation in root or shoot weight among populations, which only was present in *C. jacea*, but may partly be explained by positive correlations between the absolute abundances of different nematode feeding groups, which were especially found in nematode communities of *C. stoebe*, and in southern soil only. The mechanisms underlying these positive correlations are unknown, but one possible explanation is that southern soil contained root-feeding nematodes that positively affected the abundances of other nematode feeding groups by boosting soil microbial activity through increased leakage of nutrients from plant roots (Bardgett et al. 1999; Haase et al. 2007).

We conclude that populations from the expanded range of *C. stoebe* did not accumulate fewer root-feeding nematodes than populations from the original range, but that populations of the same plant species may vary in the accumulation of nematode feeding groups as well as root-feeding nematode types. Future studies that examine non-native plant species with known belowground specialists and generalist enemies might shed new light on the question whether shifts in defence against belowground enemies between native and non-native ranges may occur in other plant species (Huang et al. 2018; Lin et al. 2015). Finally, our study suggests that *C. stoebe* may benefit from a reduction of root-feeding nematodes in new range soil. Together, our results show that nematode community responses to plants are highly plant-species specific and geographically variable.

## Electronic supplementary material

Below is the link to the electronic supplementary material.

Supplementary material 1 (DOCX 1250 kb)

## Data Availability

Data belonging to this study are available online: 10.5061/dryad.2280gb5q5.
