# Supplementary material for: Plant population and soil origin effects on rhizosphere nematode community composition of a range-expanding plant species and a native congener
Source: Oecologia. 2020 Oct 3;194(1):237–50. doi: 10.1007/s00442-020-04749-y (PMC7561541; doi:10.1007/s00442-020-04749-y)
Supplement: Supplementary file 1 — Supplementary material 1 (DOCX 1250 kb) [file 442_2020_4749_MOESM1_ESM.docx]

Electronic supplemental material accompanying the manuscript ‘*Plant population and soil origin effects on rhizosphere nematode community composition of a range-expanding plant species and a native congener*’

**Supplementary Table 1**: seed collection sites of populations of native *Centaurea jacea* and range-expanding *Centaurea stoebe* in the Netherlands (NL) and Slovenia (SLO), including the name of the area or municipality and GPS-coordinates.

| **Species** | **Population** | **Country** | **Name area** | **Coordinates** |
| --- | --- | --- | --- | --- |
| *Centaurea jacea* | 1 | NL | Millingerwaard | 51°52'45.8"; 6°00'16.9" |
| *Centaurea jacea* | 2 | NL | Kekerdom | 51°51'55.68"; 6°0'44.37" |
| *Centaurea jacea* | 3 | NL | Kaliwaal | 51°52'04.9"; 5°59'32.5" |
| *Centaurea jacea* | 1 | SLO | Domzale | 46°08'13.54"; 014°36'58.32" |
| *Centaurea jacea* | 2 | SLO | Domzale | 46°09'91.62"; 014°45'33.90" |
| *Centaurea jacea* | 3 | SLO | Hrase | 46°22'37.64"; 014°10'06.62" |
| *Centaurea stoebe* | 1 | NL | Schinveld | 50°58'19.2"; 5°59'16.8" |
| *Centaurea stoebe* | 2 | NL | Hellevoetsluis | 51°49'54.7"; 4°7'54.5" |
| *Centaurea stoebe* | 3 | NL | Steenfabriek | 51°51'60.3"; 5°53'33.6" |
| *Centaurea stoebe* | 1 | SLO | Zadovinek | 45°55'58.10"; 015°29'78.10" |
| *Centaurea stoebe* | 2 | SLO | Zadovinek | 45°55'73.46"; 015°30'50.88" |
| *Centaurea stoebe* | 3 | SLO | Zadovinek | 45°55'82.28"; 015°29'74.02" |

**Supplementary figures**


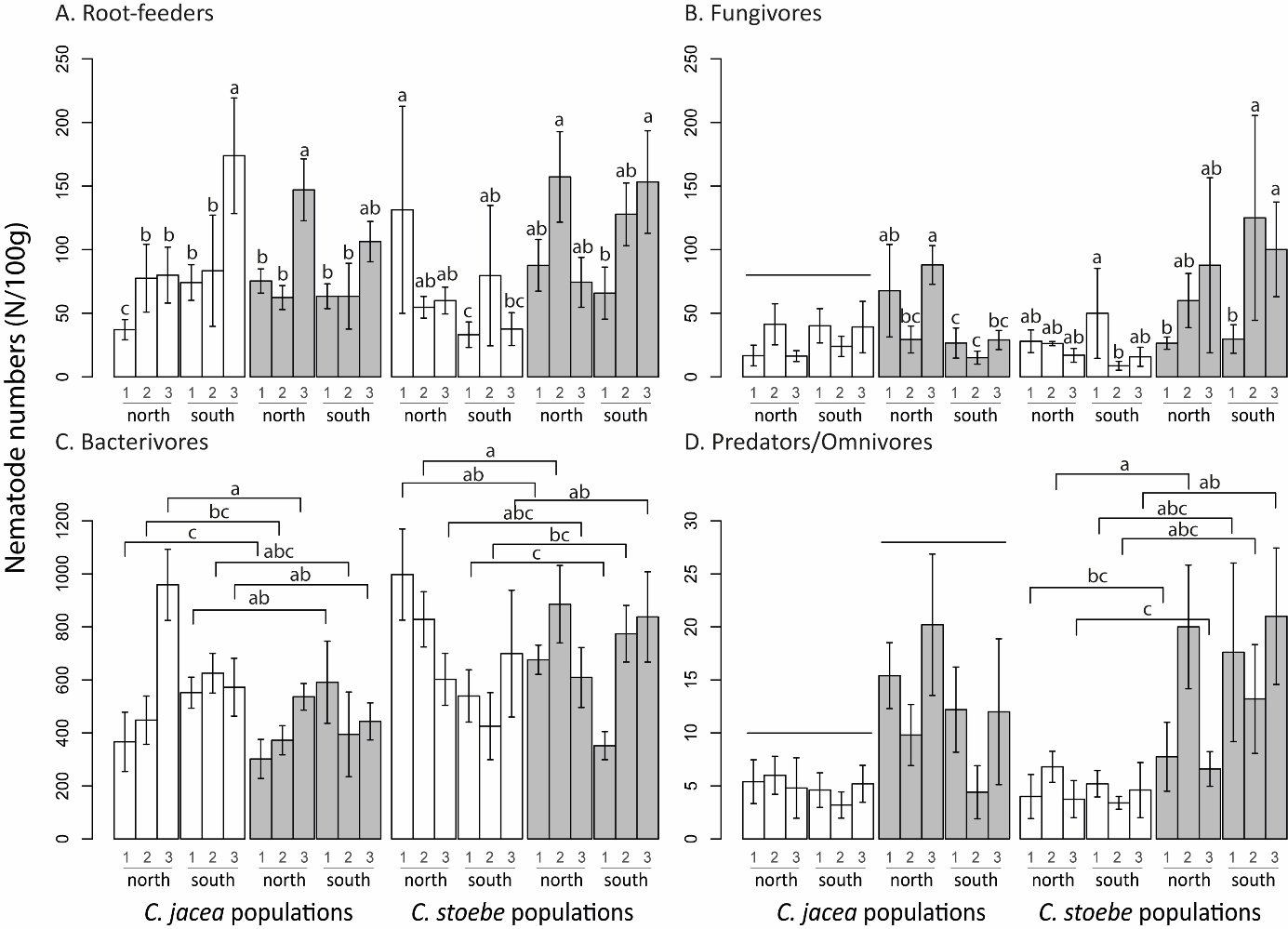


Fig. S1: Absolute abundances of the four main nematode feeding groups in three northern and three southern populations of native plant species *C. jacea* and range-expanding plant species *C. stoebe*, in northern (white) and southern soil (grey). Bars represent means ± standard errors. Horizontal brackets with small letters indicate differences between populations based on post-hoc analysis. Horizontal bars indicate absence of population effects, while individual small letters represent within-species interactions between soil and population.


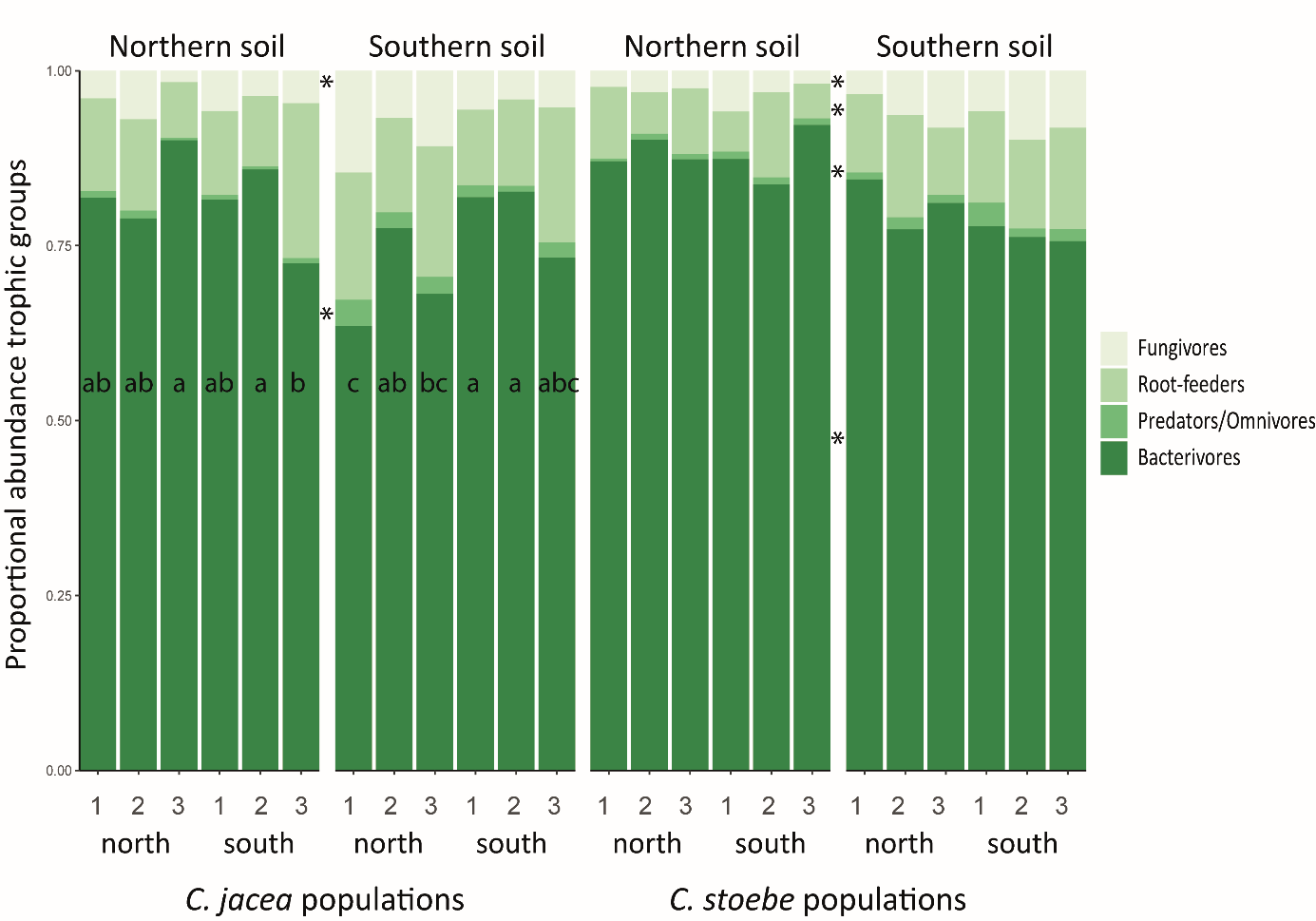


Fig. S2: Proportional abundances of the four main nematode feeding groups in three northern and three southern populations of native plant species *C. jacea* and range-expanding plant species *C. stoebe*, in northern and southern soil. Bars represent means ± standard errors. Asterisks (*) indicate significant differences between soils within species, and small letters indicate within-species interactive effects between soil origin and population.


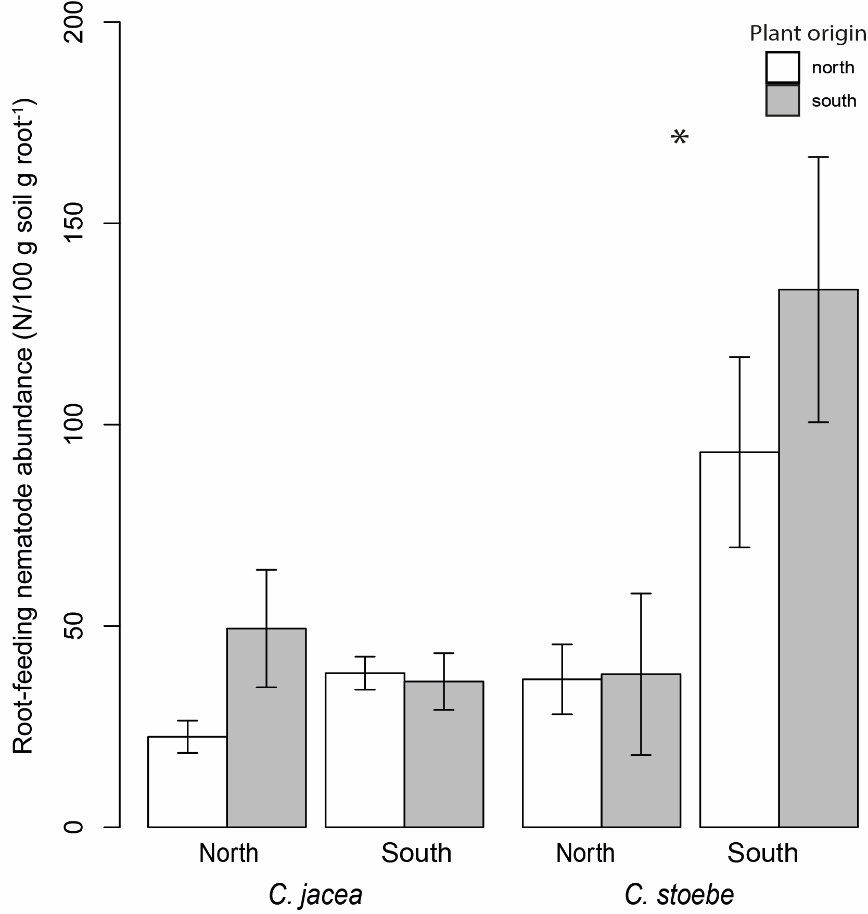


Fig. S3 Root-feeding nematode abundances adjusted for root biomass ((N/100 g soil)/root biomass)) in plants from northern (white) and southern populations (grey) of the native plant species *C. jacea* and the range-expanding plant species *C. stoebe*, in northern and southern soil. Bars represent means ± standard errors. Asterisks (*) indicate significant differences between soils within species.


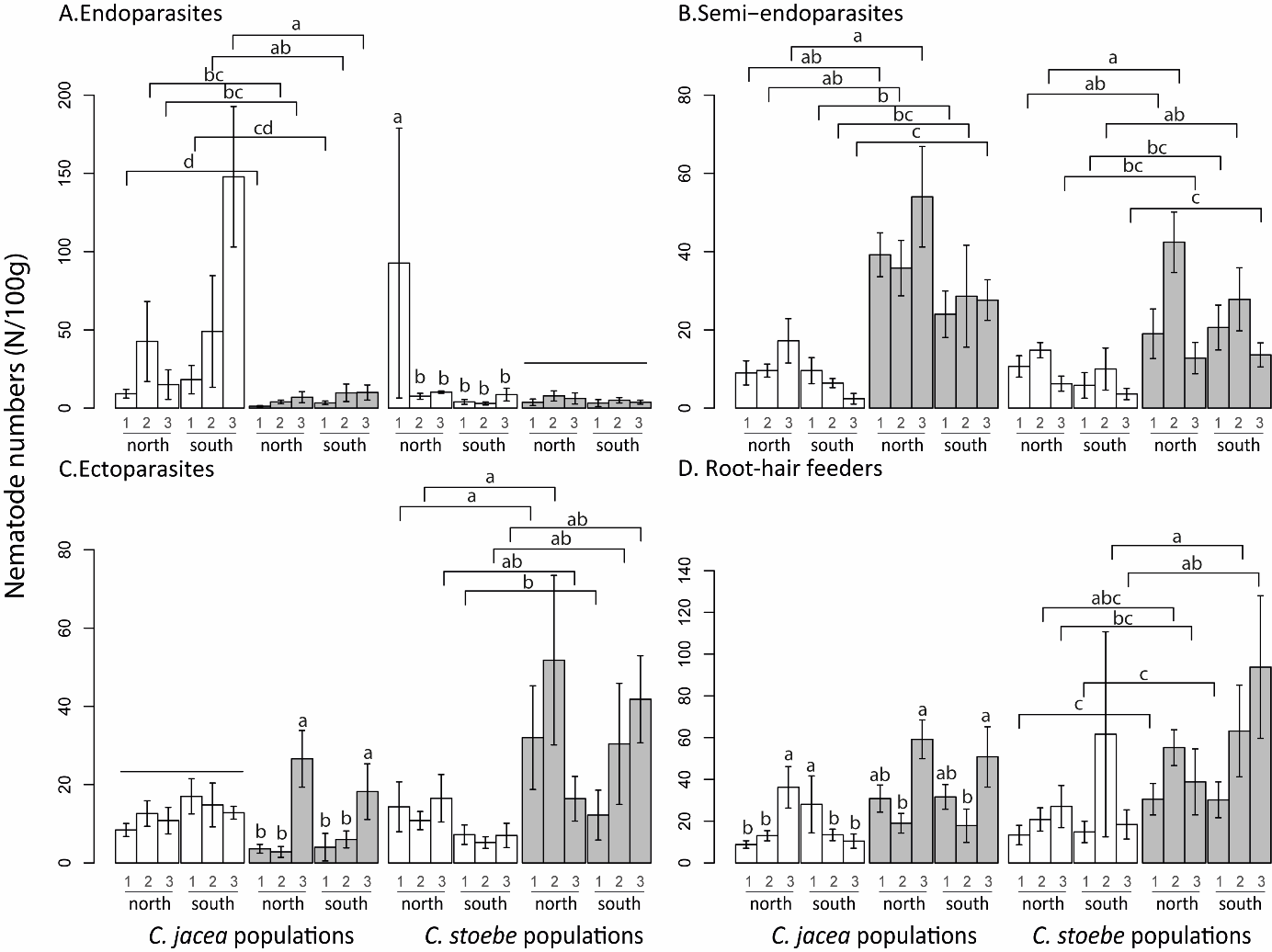


Fig. S4: Absolute abundances of the four root-feeding nematode types in three northern and three southern populations of native plant species *C. jacea* and range-expanding plant species *C. stoebe*, in northern (white) and southern soil (grey). Bars represent means ± standard errors. Horizontal brackets with small letters indicate differences between populations. Horizontal bars and small letters represent interactions between soil origin and population.
